# Quality-Adjusted Life Years in Erythropoietic Protoporphyria and Other Rare Diseases: A Patient-Initiated EQ-5D Feasibility Study

**DOI:** 10.3390/ijerph20075296

**Published:** 2023-03-28

**Authors:** Jasmin Barman-Aksözen, Anna-Elisabeth Minder, Francesca Granata, Mårten Pettersson, Cornelia Dechant, Mehmet Hakan Aksözen, Rocco Falchetto

**Affiliations:** 1 International Porphyria Patient Network (IPPN), 8032 Zurich, Switzerland; 2 Institute of Laboratory Medicine, Municipal Hospital Zurich Triemli, 8063 Zurich, Switzerland; 3 Swiss Reference Centre for Porphyrias, Municipal Hospital Zurich Triemli, 8063 Zurich, Switzerland; 4 Division for Endocrinology, Diabetology and Porphyria, Municipal Hospital Zurich Triemli, 8063 Zurich, Switzerland; 5 Fondazione IRCCS Ca’ Granda Ospedale Maggiore Policlinico, S.C Medicine and Metabolic Disorders, 20122 Milano, Italy

**Keywords:** quality of life, EQ-5D, quality-adjusted life years, erythropoietic protoporphyria, orphan drug, afamelanotide, National Institute for Health and Care Excellence, highly specialised technologies, health technology assessment

## Abstract

Erythropoietic protoporphyria (EPP) is an ultra-rare inborn error of metabolism characterised by painful phototoxic burn injuries after short exposure times to visible light. Patients with EPP are highly adapted to their condition which makes the quantification of their health-related quality of life (QoL) challenging. In the presented patient-initiated feasibility study, we describe a new approach to assess treatment benefits in EPP by measuring QoL with the generic EQ-5D instrument in five patients under long-term (≥two years) treatment with afamelanotide, the first approved therapy for EPP. For the study, we selected patients with EPP who in addition were affected by an involuntary treatment interruption (caused by a temporary reimbursement suspension) because we hypothesized that individuals who had previously unlearned their adaptation are better able to assess their life without treatment than treatment-naïve patients. QoL under treatment was comparable to the age-matched population norm, and retrospective results for a treatment interruption and phototoxic reaction time point were comparable to the QoL of patients with chronic neuropathic pain and acute burn injuries, respectively. The results were accepted by the National Institute for Health and Care Excellence in England for their evaluation of the cost-effectiveness of afamelanotide, i.e., the calculation of quality-adjusted life years.

## 1. Introduction 

Erythropoietic protoporphyria (EPP) is an ultra-rare inborn error of the heme biosynthesis (prevalence 1:100,000) that leads to the accumulation of the phototoxic heme precursor protoporphyrin during the maturation of the red blood cells [1,2,3]. Patients affected by EPP suffer from phototoxic burn injuries and inflammation of their blood capillaries in tissues exposed to visible light, i.e., sunlight and certain artificial light sources [4,5,6,7]. The associated severe neuropathic pain can last for several days and is unresponsive to pain treatment [8]. From early childhood on, patients with EPP develop a fear of exposure to sunlight and modify their behaviour and social interactions, as well as educational and professional plans according to their condition [7,8]. However, complete avoidance of light exposure is not possible and adaptations such as the cessation of voluntary outdoor activities and makeshift light protection measures, such as umbrellas and long-sleeved garments, are of limited effectiveness and subject the patients to social isolation, stigmatisation, allegations of malingering, and mental health challenges [7,8,9,10,11,12,13,14]. 

In 2014, the orphan drug afamelanotide (Scenesse^®^), the first effective treatment for the prevention of phototoxic reactions in EPP, was approved for the treatment of adult patients with EPP in the EU, followed by the USA and Australia [15]. Afamelanotide is currently available to patients with EPP in several European countries and in Israel, China and the USA (Table S1). Patients under treatment with afamelanotide reported a self-perceived near normalisation of all aspects of their daily lives and being able to expose themselves to sunlight without incurring phototoxic reactions for up to several hours a day compared with the median 10 to 15 min without the treatment [7,16,17,18,19,20]. In line with these clinical findings, the quality of life (QoL) of patients with EPP as assessed with the disease-specific EPP-QoL tool is low at baseline, and increases statistically significantly under treatment with afamelanotide, as demonstrated in several clinical trials and long-term observational studies [7,16,18,19,21]. The EPP-QoL is the only validated tool to assess QoL in EPP but has been criticised for mainly asking about disease-specific limitations [22,23]. In contrast, measurements of the QoL with the generic tools Short Form 36 (SF-36) and Dermatology Quality of Life Index (DLQI) that have been used in some of the randomised controlled clinical trials resulted in high baseline values and only small increases in QoL under treatment [21,24].

Lack of responsiveness of generic QoL tools to disease characteristics and treatment effects and unreasonably high baseline QoL measurements have been previously described for several rare and chronic conditions and/or in conditions associated with a high degree of adaptation [25,26,27,28,29]. In the presented feasibility study, we describe a new approach to assess treatment benefits in EPP with the generic QoL instrument EQ-5D (EuroQol five-dimensions questionnaire [30]). The study design is partly based on guidance provided in a report of the Decision Support Unit of the University of Sheffield which conducts research for and provides advice to the National Institute for Health and Care Excellence (NICE) in England [31]. NICE evaluates the benefits and costs of new treatments and formulates recommendations as to whether they should be funded and thus available to patients in England and Wales. For their evaluation, NICE requires generic QoL data and explicitly recommends the generic EQ-5D instrument. Results obtained with the EQ-5D can be directly used to calculate quality-adjusted life years (QALYs), a combined measure for the quality and quantity of the benefit a treatment provides. Alternatively, QALYs can be calculated, for example, from results obtained with other generic QoL tools such as the DLQI, which can be transformed into EQ-5D results by using mapping (crosswalk) algorithms [32,33]. 

Afamelanotide is currently evaluated by NICE under the Highly Specialised Technologies programme for treatments for very rare diseases. In NICE’s initial assessment issued in 2018, afamelanotide was associated with a 0.33 QALY gain (based on mapped DLQI results) and was not recommended for funding. However, the evaluating NICE committee acknowledged that the benefits of the afamelanotide treatment likely had been underestimated in the clinical trials but stressed that the lack of suitable data to estimate a more appropriate QALY gain prevented an alternative assessment [34]. The International Porphyria Patient Network (IPPN) is a stakeholder in the appraisal process of afamelanotide at NICE and together with the Swiss Reference Centre for Porphyrias at the Municipal Hospital Zurich conducted the presented feasibility study with the objectives to (1) evaluate the appropriateness of the EQ-5D instrument to measure QoL in EPP and (2) assess how the EQ-5D instrument has been used for the calculation of QALYs for treatments for very rare diseases previously assessed by NICE.

## 2. Material and Methods

### 2.1. Study Design and Cohort 

Some guidance for EQ-5D data collection is provided by the NICE Decision Support Unit report “Measuring and valuing health-related Quality of Life when sufficient EQ-5D data is not available” issued in 2020 by the University of Sheffield [31]. The report provides an overview of alternative methods accepted in previous evaluations at NICE and concludes that the collection of EQ-5D data remains challenging for some conditions, for example, in case they are associated with a high degree of adaptation or in small populations. The authors suggest that data obtained directly from the patients or caregivers should be preferred over indirect or proxy data usage, i.e., utilities from other studies or conditions, or obtained by vignette studies (in which specialised clinicians, patients, or members of the public rate descriptions of different disease states using the EQ-5D instrument). 

For our study, the participants were asked to complete the generic EQ-5D-5L and the validated, disease-specific instrument EPP-QoL for their current situation under treatment with afamelanotide and, retrospectively, for a representative day during their treatment interruption phase as well as a representative day on which they had suffered from a phototoxic reaction. Afamelanotide is currently not approved in Switzerland but patients with severe and chronically debilitating conditions can access treatments that have been approved in countries with a comparable regulatory system (e.g., EU, USA) by an individual reimbursement agreement with their health insurer (Table S1). The agreement is reviewed annually and, in 2016, many health insurers denied access to afamelanotide even for patients who had benefited from the treatment for a prolonged time. Since 2017, most patients with EPP in Switzerland have had access to treatment with afamelanotide. 

The reliability of the retrospective data for the treatment interruption was tested by comparison of the EPP-QoL data collected during our study with that from the clinical records. We further collected data on patient characteristics in a survey that was supplemented with information from the clinical records, in accordance with the informed consent. Additional data collected for this study were: age at first symptoms, age at diagnosis, and the self-assessed effectiveness of afamelanotide and the best alternative treatment on a visual analogue scale (VAS) from 0 (no effect) to 10 (best possible effect). Further, the participants were invited to share their insights and provide feedback in free comment sections and orally throughout the entire study period. 

To not compromise the identity of the participants of this study, only mean and SD values are provided in the results in Section 3. The study was conducted between July and August 2020 and the data for the study were collected once during this period. 

### 2.2. Recruitment of Study Participants

Patients with EPP are highly adapted to their condition and report that it took them a long time, i.e., several weeks to months, to overcome their anxiety and unlearn their light avoidance behaviours. Therefore, for our study, we included five patients with EPP under long-term treatment with afamelanotide, defined as ≥two years of treatment, who in addition were affected by an involuntary treatment interruption (caused by a temporary reimbursement suspension). We hypothesized that individuals who had previously unlearned their adaptation can assess their life without treatment better than treatment-naïve patients who might have no perception of a normal light exposure. As the retrospective study questions were assessed as having the potential to reactivate traumatic memories (such as loss of employment, depressive symptoms, and suicidal ideations), we limited the participants of this feasibility study to a maximum of five patients. Patients with EPP from the Swiss Reference Centre for Porphyrias were recruited for this study after written consent was obtained and were required to meet all of the following criteria: (1) prior written consent to the ongoing biobank research project at the porphyria outpatient clinic at the Municipal Hospital Zurich (approval number of the cantonal ethics committee, Zurich: BASEC 2018–00758); (2) long-term treatment experience with afamelanotide defined as ≥two years and currently under treatment; (3) having been affected by an afamelanotide treatment interruption of ≥1 month between 2 doses (afamelanotide is administered as a slow-release implant formulation every 60 days prior to expected and during increased light exposure); (4) emotional stability as assessed by a physician familiar with EPP to minimize the risk for reactivation of traumatic memories by the retrospective study questions; and (5) being a member of a patient organisation or other support group in case the study induces stress and the participants feel the need to exchange with peers. Patients involved in the planning or execution of the study were excluded from participation as trial subjects. 

In total, 20 Swiss patients were affected by an involuntary treatment interruption in 2016, and 11 individuals met all inclusion criteria for our study. One of the patients initially invited did not want to participate in the study, with the reasons given being time constraints and not wanting to recall the period with the treatment interruption. 

### 2.3. The EQ-5D Instrument 

The generic EQ-5D (EuroQol five-dimensions questionnaire) instrument contains five questions for the five QoL dimensions “mobility”, “self-care”, “usual activities”, “pain/discomfort”, and “anxiety/depression” [30]. The 5L-version of the EQ-5D questionnaire used for this study contains five grading levels with one being the best possible and five the worst possible health state, while the original EQ-5D-3L version contains three grading levels, respectively. In addition, the questionnaire contains the Visual Analogue Scale EQ-VAS, ranging from 0 (worst possible state) to 100 (best possible state). The default recall period of the EQ-5D is “today”. For the retrospective time points of our study, the recall period of the EQ-5D was rendered to “a representative day during the treatment interruption” and “a representative day during a phototoxic reaction”, respectively. The EQ-5D has been validated for several conditions, but not for EPP [35]. 

### 2.4. Calculation of Quality-Adjusted Life Years

Quality-adjusted life years (QALYs) are a combined measure for the quality and quantity of the benefit an intervention provides. It can be used to compare treatments for the same disease or across disease areas. The EQ-5D is one of the most frequently used instruments to obtain QALYs. The results (profiles) of the EQ-5D instruments correspond to preference-based health index values (utilities) in previously established national value sets. In general, a health index value of 1 is the best possible outcome, and 0 is considered being dead. Because no value set has been established for Switzerland so far, in accordance with the EQ-5D-5L user guide we used the German value set and the crosswalk algorithm published by van Hout et al. [36] to calculate the health index values. The age-matched German population norms for the health index value (0.962) and the EQ-VAS scale (82.5) were retrieved from Szende et al. [37]. For the calculation of the QALYs gain, further assumptions are required, such as the length of the treatment and experienced benefits (time horizon), and discount rates on the benefits. In this manuscript, QALYs outcomes are reported as discounted with the standard 3.5% rate, if not stated otherwise. 

### 2.5. The EPP-QoL Instrument 

The disease-specific EPP-QoL questionnaire was developed by clinical experts and the sponsor of the clinical trials evaluating afamelanotide because of feedback from the patients that, in their assessment, the generic instruments used in some of the randomised controlled trials (RCTs) testing afamelanotide (i.e., the SF-36 and DLQI) did not adequately capture the disease characteristics and burden of the condition and did not reflect the treatment effects. The final questions were developed including insights provided by patients with EPP. The validation of the EPP-QoL was ongoing during the clinical trials and completed in 2021 [23]. The final version of the EPP-QoL has 12 items and 4 grading levels. The best possible QoL outcome is 100%. The default recall period of the EPP-QoL is the last two months. The EPP-QoL is currently the only validated tool for the evaluation of QoL in EPP. 

### 2.6. Reliability of the Retrospective Data

Patients at the porphyria outpatient clinic of the Municipal Hospital Zurich complete the EPP-QoL questionnaire every two months during their appointments to receive their treatment with afamelanotide. To measure the reliability of the data collected retrospectively for this study, we compared the EPP-QoL results obtained in our study with that from the clinical records collected directly after the treatment interruption and in 2018 when the patients were under treatment again. 

### 2.7. Appropriateness of the Questionnaires

To evaluate how well the questions of the two instruments reflect aspects relevant to the patients, we asked the participants to rate the questions in the EQ-5D-5L and EPP-QoL questionnaires according to their appropriateness for assessing EPP disease characteristics for the three time points “under treatment”, “treatment interruption”, and “phototoxic reaction”. The participants could rate the single questions as being “very appropriate”, “appropriate”, “less appropriate”, and “not appropriate”. The answers were scored on a scale from one (very appropriate) to four (not appropriate). In addition, the participants could provide free comments regarding the appropriateness of the questions. 

### 2.8. Statistical Analysis 

For correlations, Pearson’s r was performed. A correlation coefficient r of 0.4–0.69 was considered as moderate, 0.7–0.89 as strong, and ≥0.9 as a very strong correlation. As our study only had five participants, we did not deem it feasible to conduct further statistical analyses.

### 2.9. EQ-5D and QALY Gains in Highly Specialised Technologies Previously Assessed at NICE 

In 2013, NICE introduced the Highly Specialised Technologies (HST) programme, a separate pathway for the evaluation of treatments for very rare conditions which allows the evaluating committee to consider a wider range of evidence, for example, data derived from non-RCT or natural history studies. Further, since 2017, highly specialised technologies that produce an undiscounted QALY gain between 10 to 30 QALYs over the lifetime of a patient are considered highly effective and are multiplied (weighted) by a factor between 1 to a maximum of 3, which increases their likelihood for a positive recommendation for funding by NICE [38].

As NICE explicitly recommends the EQ-5D instrument for measuring QoL, we analysed which instruments have been employed in the clinical trial testing treatments evaluated under the HST programme and how these results were used for the QALY calculation. Therefore, from the NICE website we retrieved the evaluation documents (Final Evaluation Determination documents, Committee Papers, Public Committee Meeting slides and the Evidence Review Group reports) for all concluded evaluations of highly specialised technologies from the start of the programme in 2013 until December 2022. We analysed the sections of the documents describing the assessment of the QoL data, in particular the specifics of the QALY calculation such as the assumed time horizon and discount rates on costs and benefits, and the resulting QALY gains. 

### 2.10. Researcher Characteristics 

The International Porphyria Patient Network (IPPN) is a not-for-profit organisation of patients and carers that provides support and counselling for patients suffering from one of the eight forms of porphyria and collaborates with national and international porphyria patient associations in scientific, medical, and healthcare policy matters. The IPPN is a stakeholder at NICE in the appraisal proceedings of the highly specialised technologies afamelanotide for treating erythropoietic protoporphyria (ID927) and givosiran for treating acute hepatic porphyria (HST16). 

## 3. Results

All results are given in means (SD), if not stated otherwise 

### 3.1. Patient Characteristics Data 

Patient characteristics data of the investigated individuals are given in Table 1. The obtained patient characteristics data were within the range of the Swiss and international cohorts [7,16,18,19,20]. 

### 3.2. QoL in EPP as Determined with the EQ-5D Instrument

**Figure 1:**
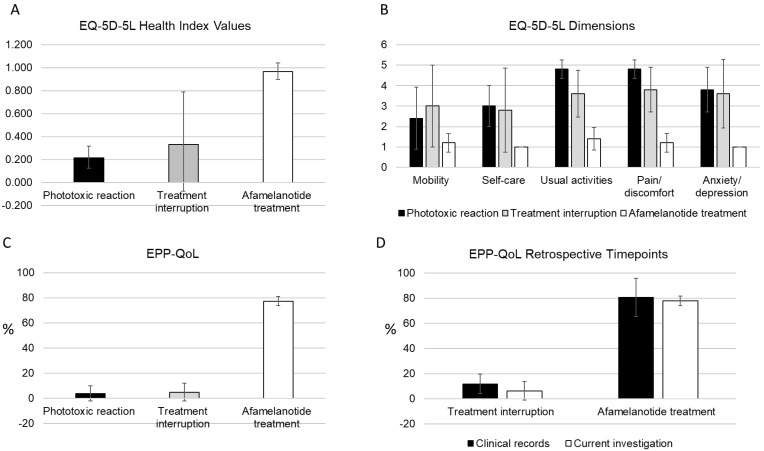
(**A**–**D**): Results obtained with the EQ-5D and EPP-QoL instruments for patients with erythropoietic protoporphyria.

The EQ-5D health index value under treatment with afamelanotide in our cohort was 0.965 ± 0.08. The retrospective EQ-5D values for the treatment interruption and the phototoxic reaction time points were 0.331 ± 0.46 and 0.215 ± 0.10, respectively (Figure 1A). The results for the five EQ-5D dimensions are given in Figure 1B. Under treatment, the mean value for the 5 dimensions was 1.2 ± 0.4, during the treatment interruption 3.4 ± 1.6, and during the phototoxic reaction 3.8 ± 1.3, respectively. The EQ-VAS scale was 89 ± 4.2 under treatment and 28 ± 25.6 and 15 ± 8.7 for the treatment interruption and phototoxic reaction time points, respectively. 

### 3.3. QoL in EPP as Determined with the EPP-QoL Questionnaire

The QoL results obtained with the EPP-QoL instrument were 77.2% ± 3.6% under treatment, 5.0% ± 6.9% during the treatment interruption, and 3.9% ± 6.1% for the phototoxic reaction, respectively (Figure 1C). 

### 3.4. Reliability of the Retrospective Data 

For four patients, EPP-QoL results from the medical records were available for the afamelanotide treatment and treatment interruption time points. At the end of 2018, when all patients in Switzerland were again under treatment with afamelanotide, the QoL in these 4 patients was 80.6% ± 15.2%. The same individuals assessed their QoL in July 2020 as being 77.8% ± 3.9%. The QoL from the medical records collected during the treatment interruption between 2016 and 2017 was 11.8% ± 7.6%. The same individuals retrospectively assessed their QoL for this period as being 6.3% ± 7.3% (Figure 1D).

### 3.5. Correlation between the QoL Instruments and the EQ-VAS Scale

The correlation between the results obtained with the EQ-5D-5L (health index values) and the results of the EQ-VAS was r = 0.961. The correlation between the results of the EQ-5D-5L and the results of the EPP-QoL was r = 0.833. Further, the correlation between the EPP-QoL results and the EQ-VAS values was r = 0.931. 

### 3.6. Appropriateness of the Questionnaires 

Four patients returned their ratings about the appropriateness of the questions of the EQ-5D-5L and the EPP-QoL. On average, the EPP-QoL instrument was rated as being “very appropriate” to capture disease characteristics and treatment effects in EPP during the afamelanotide treatment and the treatment interruption phase but less appropriate for the phototoxic reaction. In the free comment sections, the participants suggested that the psychological aspects of EPP should be better captured. They appreciated that the questions also cover problems caused by artificial light and stressed that having to plan the day is an important aspect in EPP that is only recognized in the EPP-QoL.

On average, the EQ-5D-5L was rated as being “appropriate” to measure disease characteristics of EPP for all three time points (treatment, treatment interruption, and phototoxic reaction). When the dimensions were assessed independently, mobility and self-care were rated as being less appropriate, while usual activities, pain/discomfort, and anxiety/depression, were rated as being very appropriate questions. However, one patient shared that while travelling during her summer holidays she fractured her leg. As the EQ-5D questionnaire does not distinguish between EPP-related symptoms and pain or mobility in general, the results would have been misleading when assessing treatment benefits of afamelanotide with this instrument during this time in which she was under treatment and did not suffer from any symptoms caused by her EPP. In the free comment section, the participants also explained that the mobility dimension is ambiguous (they might be perfectly able to walk about, but nevertheless they cannot leave the house because the sun shines) and that, in general, they would appreciate more EPP-specific wording of the questions. 

An aspect neither covered by the EPP-QoL nor by the EQ-5D-5L is the severe fatigue during and after a phototoxic reaction, which compromises the ability to function for up to two weeks. 

### 3.7. EQ-5D Data in Previously Assessed Highly Specialised Technologies 

**Figure 2:**
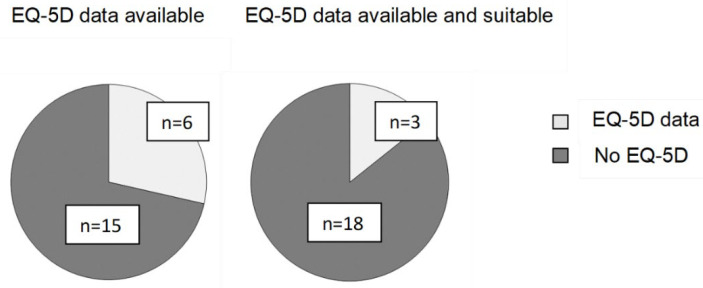
A (EQ-5D data available)+ B (EQ-5D data available and suitable): EQ-5D collected in clinical trials or observational studies in the evaluation of previously assessed highly specialised technologies at the National Institute for Health and Care Excellence in England (Table S2).

Since the start of the HST programme in 2013 until December 2022, evaluations for 20 highly specialised technologies (HST 1–18, 20, 21) were concluded, which all received a positive recommendation for funding (Table S2). For five of these technologies, EQ-5D data were measured in the clinical trials, i.e., HST 1, 10, 12, 13, and 16 (Figure 2A). However, only in 2 of these cases (HST 1 and 10), this data were also used for the calculation of the QALY gains that informed the decisions of the HST committee. In the remaining three cases, the EQ-5D data collected during the clinical trials were assessed as not being suitable for calculating the QALY gain (for example, because the evaluating committee assessed that the condition was associated with a high degree of adaptation and that the duration of the trials was too short to demonstrate the treatment benefits). In addition, data collected during a managed access agreement for the previously evaluated technology HST 2 were reviewed (HST 19) and the technology again recommended for use in the National Health Service in England and Wales. The EQ-5D data collected during the managed access agreement were used for the review of HST 2. 

Therefore, only for 3 (14.3%) of the 21 evaluations of highly specialised technologies, EQ-5D data were collected during the clinical trials or observational studies and assessed as being suitable (Figure 2B). In nine evaluations, the QALY gain was calculated based on data from the literature, either from the same or a proxy condition (assumed to have comparable disease characteristics and/or treatment effects). For the remaining nine evaluations, separate vignette studies were performed. 

### 3.8. Time Horizons in Previously Assessed Highly Specialised Technologies

For the calculation of QALYs, the time over which the benefits are accumulated needs to be considered. For 18 of the highly specialised technologies, the time horizons used for the QALY calculation were reported in the appraisal documents and ranged from 35 to 125 [*sic*] years, with a median of 100 years (mean 81.8 ± 27.5 years) (Table S3). Shorter time horizons usually corresponded to the estimated maximum remaining lifetime for the particular condition, for example, in the case of a disease that only presents later in life. For the QALY calculation of afamelanotide, a time horizon of 35 years was used (Table 2). EPP usually first presents in early childhood and, with the exception of rare complications affecting the liver, is not associated with a reduced life expectancy.

### 3.9. QALY Gains and Discount Rates in Previously Assessed Highly Specialised Technologies

QALY gains and discount rates of highly specialised technologies are reported in the Final Evaluation Determination documents available from the NICE homepage (Table S4A–C). A discount rate of 3.5% for costs and benefits over the assumed time horizon is considered the standard rate by NICE. For highly specialised technologies, a discount rate of 1.5% can be accepted by the committee in case the treatment is expected to restore the health of people who would otherwise die or have a severely impaired life and if the effect is sustained for a period of at least 30 years. In the Final Evaluation Determination documents, the information on QALY gains is provided as either discounted with a rate of 3.5%, or a rate of 1.5%, or as undiscounted QALY gains. Further, for 12 (57.1%) of the 21 evaluations, the QALY gains are only reported as ranges, for example “≥10 undiscounted QALYs” because they were considered commercial in confidence by the manufacturers. To better reflect the characteristics of the different technologies, we divided the highly specialised technologies into three different categories, i.e., technologies tested in non-inferiority trials, gene therapies, and other technologies. A total of 2 evaluations (HST 4 and HST 5) concerned new technologies (oral formulations) which were tested in non-inferiority trials against the standard of care (infusions) assessed as having the same efficacy (Table S4A). For these treatments, QALY gains (discounted with 3.5%) of 0.34 and 1.05 are reported, respectively. A total of 4 evaluations (HST 7, 11, 15, and 18) concerned gene therapies, with reported undiscounted QALY gains between ≥10 to ≥30 (Table S4B). The remaining 15 technologies were associated with QALY gains between >3.05 (discounted with a rate of 3.5%) to ≥30 undiscounted QALYs (Table S4C). For afamelanotide, a lifetime undiscounted QALY gain of 0.56 (0.33 when discounted with 3.5%) was the preferred estimate of the committee (Table 2).

## 4. Discussion

In our feasibility study, measurement of the QoL with the generic instrument EQ-5D in five patients with EPP under long-term treatment with afamelanotide resulted in health index values and corresponding EQ-VAS scores slightly above the age-matched population norms (Figure 1A) [37]. Therefore, the results as measured with the EQ-5D instrument correspond to the subjective improvements and the near-normalisation of their daily lives as reported by the study participants and reflected in their patient characteristics data (Table 1). The health index values for a phototoxic reaction collected retrospectively in our cohort were comparable to values found in people with acute burn injuries at the time of their hospital admission [39,40,41]. In our assessment, these health index values are plausible because the pain associated with a phototoxic burn injury in EPP is described as devastating, long-lasting, excruciating, and able to elicit suicidal ideations [8,20]. The measured mean health index value for a representative time during a period with treatment interruption was comparable to values reported, for example, from people with chronic neuropathic pain [42]. However, the variation in health index values obtained from our cohort was considerably higher. The degree of impairment during a treatment interruption depends largely on external circumstances, for example, the requirement to commute to work or whether the patient has a home office solution. Interestingly, the variation in results as measured with the disease-specific instrument EPP-QoL were less pronounced, most likely because the questions are more specific to the disease characteristics and treatment effects. 

The participants of our study assessed the EQ-5D questionnaire as being an overall “appropriate” instrument to measure the burden of the EPP condition and treatment effects. However, the disease-specific instrument EPP-QoL was assessed as “very appropriate” and more sensitive to the EPP disease characteristics. For example, pain or restrictions in mobility caused by unrelated health issues, for example, a fractured leg, can confound QoL results when measured with generic instruments, in contrast to the disease-specific EPP-QoL questionnaire.

An apparent limitation of our study is the small sample size of only five participants. The patient characteristics data collected for this study suggest that the severity of the disease and extent of treatment benefits seen in the included individuals are within the range that is observed for cohorts from international treatment centres, thus mitigating concerns of an unintentional selection bias of our study cohort [7,16,18,19,20]. For example, a recent study from The Netherlands measuring QoL with, amongst other tools, the generic SF-36 instrument in the Dutch cohort (n = 121) demonstrates improvements in several domains such as social function and pain, in line with our measurements [7]. Nevertheless, the results of our study should be confirmed in bigger cohorts with the EQ-5D instrument.

Another main limitation of our study is that the data for the phototoxic reaction and the treatment interruption time points were collected retrospectively. The default recall period of the EQ-5D is “today” and the instrument is not validated for retrospective assessments. In research conducted on injury-related disabilities, e.g., burn victims and survivors of accidents, prospectively collected baseline QoL data are usually not available. Therefore, to assess the baseline QoL, generic instruments, including the EQ-5D, were applied retrospectively to establish the status before the injury for periods of up to 18 months, while the recall reliability was tested in repeated retrospective measurements [43,44]. To assess the reliability of the results of the retrospective time points in our cohort, we compared data collected in 2018 with the EPP-QoL from the medical records of the participants with EPP-QoL data generated during our study (Figure 1D). Our results suggest that the patients included in our study accurately recalled their QoL for the treatment interruption period, as previously observed for other conditions with major disease impacts [45]. Moreover, the time point in 2018 was prior to the COVID-19 pandemic and, therefore, in addition, can serve as a reference point to control for any confounding effects on QoL measurements caused by the pandemic.

A strength of our study is that it was conducted without funding from the industry. Because the study was initiated by patients and the main ideas originate from members of the IPPN, aspects relevant to patients with EPP are considered in the design. Further, the study represents a new approach to dealing with methodological challenges associated with adaptation in rare and chronic diseases highlighted by, for example, the DSU report [31].

In NICE’s initial assessment issued in 2018, afamelanotide was associated with an 0.33 QALY gain (discounted with 3.5%, based on mapped DLQI results from the clinical trials) and not recommended for funding (Table 2). However, the evaluating NICE committee acknowledged that the benefits of the afamelanotide treatment as measured with the generic QoL instruments likely had been underestimated but stressed that the lack of suitable data to estimate a more appropriate QALY gain prevented an alternative assessment [34]. Therefore, although associated with limitations as detailed above, during a consultation in March 2022 the IPPN shared the results obtained in this study with the evaluating HST committee. It is noteworthy that the HST committee previously accepted EQ-5D data and QALY outcomes derived from vignette studies that only included four to five clinical experts evaluating the described disease states [31]. In addition, based on comparisons with treatments for very rare diseases previously evaluated under the HST programme, the IPPN submitted suggestions on how to further improve the calculation of the QALY gain to better reflect the disease characteristics and treatment effects (Tables S2 and S3). One suggestion concerned expanding the assumed time horizon from 35 years in the initial assessment to at least 70 years, as previously used for other lifelong chronic conditions. Further, the IPPN pointed out that it lacks face validity in that the first effective treatment for EPP is associated with a gain of only 0.33 QALYs, while in the evaluation of HST 5, switching from an infusion to an oral therapy (having the same efficacy) was already associated with a 1.05 QALY gain (Table S4A). The HST committee indeed considered several of the provided insights and in their current assessment issued in September 2022 based their preferred assumption partly on data and suggestions submitted by the IPPN [46]. Currently, a 60-year time horizon is assumed and the afamelanotide treatment is associated with a 9.995 QALY gain (discounted with 3.5%, own calculation, Table 2). At the time this manuscript was drafted (January 2023), the evaluation by NICE on whether afamelanotide is considered cost-effective and should be provided to patients with EPP in England and Wales was still ongoing.

Based on the comparison with health index values measured in other conditions and QALY gains accepted for previously evaluated highly specialised technologies as discussed above, we assume that a QALY gain of 9.995 better reflects the clinical benefit of afamelanotide than the initial assessment (Table S4A–C). On the one hand, the adjustments are a rewarding outcome of the invested time and efforts for this patient-initiated research project which demonstrate the open-mindedness of the evaluating HST committee. On the other hand, the increase in QALY gains by over 30-fold raises questions regarding the reliability of the QALY method when applied to very rare and chronic conditions, such as EPP [47]. Several national authorities require the calculation of QALY gains for their cost-effectiveness evaluations (Table S1). In 2016, Dutch authorities issued a positive recommendation for reimbursement for afamelanotide. In contrast, until now, England has maintained their negative assessment. This is despite having a comparable willingness to pay threshold to The Netherlands [14,48]. In 2018, Norway based their negative recommendation for funding afamelanotide on the preliminary negative recommendation in England [49]. In contrast, in Scotland the existing evidence was assessed as sufficient to recommend access within the ultra-orphan pathway in 2021, allowing for the collection of additional real-world data for the reassessment of the decision after three years. Manifestly, the use of QALY gains, although based on the same evidence which was generated during the clinical trials, does not necessarily lead to consistent evaluations of the cost-effectiveness of treatments. We hope that the presented feasibility study enables a better assessment of the treatment benefits of afamelanotide and encourages prospective data collection with the EQ-5D instrument. However, the assessment of the baseline QoL with the EQ-5D and other generic instruments in treatment-naïve patients with EPP will remain challenging. 

Moreover, although recommended by NICE, data as measured with the EQ-5D instrument in the clinical trials was only available in 5 out of 20 evaluations of new treatments and used only in 2 cases for the final decision making (Table S2). In the other three cases, the EQ-5D data collected in the clinical trials were assessed as not being suitable for evaluation. Therefore, the requirement to provide EQ-5D data for the evaluation of the cost-effectiveness of treatments for very rare diseases at NICE should be revisited. 

## 5. Conclusions

Our feasibility study with five patients with EPP indicates that QoL can be assessed with the generic EQ-5D instrument in individuals under long-term treatment with afamelanotide, the only approved treatment for this condition. Long-term treatment, defined as ≥two years, leads to near-normalised health index values which correspond to the clinical improvements and subjective assessment of the treatment benefits by the patients. The EQ-5D questionnaire was rated as an overall appropriate instrument to measure disease characteristics and treatment effects by the patients. However, health issues not related to EPP may confound the findings, as the wording is not specific to symptoms related to EPP. Moreover, as EPP is associated with a high degree of adaptation, measuring baseline health index values remains challenging. Our analysis of concluded evaluations of highly specialised technologies by NICE suggests that the EQ-5D instrument might in general not be very suitable for the assessment of treatments for very rare diseases, as for most treatments, EQ-5D data were either not collected in the clinical trials or assessed as not being suitable for the decision making. Therefore, the requirement to provide EQ-5D data for the evaluation of the cost-effectiveness of treatments for very rare diseases at NICE should be revisited. 

## Figures and Tables

**Table 1:** Patient characteristics data. Patient characteristics data of the five participants in means (SD), if not stated otherwise.

Patient Characteristics	Results
Age [years]	41.2 (±6.9)
Age at first symptoms [years]	1.9 (±0.8)
Age at diagnosis [years]	13.6 (±9.2)
Erythrocyte-free protoporphyrin [µmol/L; ULN = 0.2]	19.5 (±5.0)
Patients with phototoxic reactions caused by artificial light [*n*]	4
Patients with phototoxic reactions in winter [*n*]	5
Patients with an EPP-related liver condition [*n*]	1
Patients with a lifetime diagnosis of depression [*n*]	3
Treatment with afamelanotide [years]	9.1 (±4.0)
Duration of treatment interruption [months]	9.1 (±8.3)
Time since interruption [months]	35 (±18.0)
Number and severity of phototoxic reactions VAS 0 (no pain)—VAS 10 (worst imaginable pain)	
Number of phototoxic reactions per year, before treatment with afamelanotide [*n*]	21.2 (±8.8)
Number of phototoxic reactions per year, under treatment with afamelanotide [*n*]	4.6 (±3.3)
Maximum pain intensity of a phototoxic reaction experienced before treatment with afamelanotide [VAS]	10 (±0)
Maximum pain intensity of a phototoxic reaction experienced under treatment with afamelanotide [VAS]	5.6 (±3.3)
Phototoxic burn tolerance time	
Maximum time in sunlight without experiencing symptoms before treatment with afamelanotide [minutes]	4.4 (±3)
Maximum time in sunlight without experiencing symptoms under treatment with afamelanotide [minutes]	252 (±78)
Phototoxic burn protection factor	87.2 (±55)
Self-assessed effectiveness of treatments VAS 0 (no effect)—VAS 10 (best possible effect)	
Afamelanotide [VAS]	8.8 (±0.7)
Best alternative [VAS]	0 (±0)

ULN—upper limit of normal; VAS—visual analogue scale.

**Table 2:** Quantification of QALYs in the assessment of afamelanotide for treating erythropoietic protoporphyria at NICE.

Afamelanotide (ID927)	Accepted Method for QALY Quantification	Utilities	Assumed Time Horizon	QALY Gain, Undiscounted	QALY Gain, Discounted
Initial assessment (2018)	DLQI data as collected in the pivotal RCT	0.016	**35 years**	**0.56**	0.33
Current assessment (2022)	EQ-5D data as collected by the IPPN, modified by NICE	0.397	**60 years**	23.796	9.995

QALY: Quality-adjusted life year, NICE: National Institute for Health and Care Excellence, DLQI: Dermatology Quality of Life Index, IPPN: International Porphyria Patient Network, and RCT: randomised controlled trial. In bold: Values provided in the Final Evaluation Determination and Evaluation Consultation documents, Committee papers, and reports of the Evidence Review Group (Table S5). Other values were calculated based on further information provided in the public documents of the NICE appraisal procedure. A standard discount rate of 3.5% for the costs and benefits of treatments evaluated by NICE was applied for QALY gains in the initial and the current assessment.

## Data Availability

Data supporting the analysis can be found at the website of the National Institute for Health and Care Excellence in England: https://www.nice.org.uk/about/what-we-do/our-programmes/nice-guidance/nice-highly-specialised-technologies-guidance (last accessed 29 January 2023).

## Supplementary Materials

The following supporting information can be downloaded at: https://www.mdpi.com/article/10.3390/ijerph20075296/s1, Table S1: Countries with access to the afamelanotide treatment; Table S2: EQ-5D data in previously assessed highly specialised technologies; Table S3: Time horizons of previously assessed highly specialised technologies; Table S4: Discount rates and QALY gains of previously assessed highly specialised technologies; Table S5: Information for the back calculation for the QALY gains.
