# Supplementary material for: Quality-Adjusted Life Years in Erythropoietic Protoporphyria and Other Rare Diseases: A Patient-Initiated EQ-5D Feasibility Study
Source: Int J Environ Res Public Health. 2023 Mar 28;20(7):5296. doi: 10.3390/ijerph20075296 (PMC10094018; doi:10.3390/ijerph20075296)
Supplement: Supplementary file 1 [file ijerph-20-05296-s001.zip › final version of Supplement.pdf]

Supplement

Quality Adjusted Life Years in erythropoietic protoporphyria and other rare diseases. A patient-initiated EQ-5D feasibility study

Jasmin Barman-Aksözen, Anna-Elisabeth Minder, Francesca Granata, Mårten Pettersson, Cornelia Dechant, Mehmet Hakan Aksözen, Rocco Falchetto

Table S1: Countries with access to the afamelanotide treatment p.2

Table S2: EQ-5D data in previously assessed highly specialised technologies p.4

Table S3: Time horizons of previously assessed highly specialised technologies p.7

Table S4: Discount rates and QALY gains of previously assessed highly specialised technologies p.10

Table S5: Information for the back-calculation for the QALY gains p.13

Table S1: Countries with access to the afamelanotide treatment

Afamelanotide is currently regularly reimbursed in several European countries, in the US and Israel. For additional countries, special access schemes exist on an individual patient base. The table provides an overview over countries with access to the afamelanotide treatment for patients with erythropoietic protoporphyria as of February 2023, in the order in which afamelanotide became available.

Country	Year afamelanotide became available	Form of reimbursement	Comment
Italy	In 2009 national marketing authorisation, followed by approval in the EU in 2014	Regular reimbursement	Since 2016: Decision for reimbursement by the regional authorities
Switzerland	2012	Special access scheme, based on approval in Italy and by the EU	Individual reimbursement agreements
The Netherlands*	2016	Regular reimbursement	In addition, individual reimbursement for patients from age 16
Germany	2017	Regular reimbursement	Limited access because of lack of treatment centres
Luxembourg	2016	Individual reimbursement agreements	Individual reimbursement for single patients
Belgium	2017	Individual reimbursement agreements	Individual reimbursement for single patients
Austria	2017	Individual reimbursement agreements	Individual reimbursement for single patients
Sweden*	2019	Individual reimbursement agreements	Individual reimbursement for single patients

USA	2020	Marketing authorisation in 2019, regular reimbursement	Access might depend on insurance scheme
China	2020	Named Patient Program	
Israel	2021	Regular reimbursement	
Scotland*	2021	Not recommended, but reimbursement within the ultra-orphan pathway	Reassessment based on data collected within the ultra-orphan pathway after three years

* Countries in which the systematic benefit assessment is based on Quality Adjusted Life Years.

Table S2: EQ-5D data in previously assessed highly specialised technologies

QoL as measured with the generic EQ-5D instrument for highly specialised technologies with concluded appraisals (until December 2022), as reported in the Final Evaluation Determination documents available from the NICE homepage. Two versions of the questionnaire are currently available for adult patients, the EQ-5D-5L which contains five grading levels and the original EQ-5D-3L with three grading levels, respectively.

HST	Technology	QoL instruments used in the clinical trials	Accepted method to calculate the QALY gain
HST1	Eculizumab for treating atypical haemolytic uraemic syndrome	- **EQ-5D**	- **EQ-5D data collected in the clinical trials**
HST2	Elosulfase alfa for treating mucopolysaccharidosis type IVa	- Disease specific instrument MPS HAQ	- Literature/ other studies
HST3	Ataluren for treating Duchenne muscular dystrophy with a nonsense mutation in the dystrophin gene	- Pediatric Quality of Life (PedsQL) inventory<br>- Paediatric Outcomes Data Collection Instrument (PODCI) - Activities of Daily Living Questionnaire	- Literature/ other studies
HST4*	Migalastat for treating Fabry disease	- Short Form-36 (SF-36) questionnaire<br>- Brief Pain Inventory short form	- Literature/ other studies
HST5*	Eliglustat for treating type 1 Gaucher disease	- SF-36<br>- Fatigue severity score (disease specific)	- Literature/ other studies
HST6	Asfotase alfa for treating paediatric-onset hypophosphatasia	- Childhood Health Assessment Questionnaire (CHAQ)<br>- Paediatric Outcome Data Collection Instrument (PODCI)<br>- Lower Extremity Functional Scale (LEFS)	- Vignette study

HST7[#]	Strimvelis for treating adenosine deaminase deficiency–severe combined immunodeficiency	- PedsQL<br>- Lansky Performance Status Index	- Literature/ other studies
HST8	Burosumab for treating X-linked hypophosphataemia in children and young people	- SF-36	- Vignette study
HST9	Inotersen for treating hereditary transthyretin amyloidosis	- Modified Neuropathy Impairment Score +7 (mNIS+7)<br>- Norfolk Quality of Life-Diabetic Neuropathy (Norfolk QoL-DN) questionnaire	- Literature/ other studies
HST10	Patisiran for treating hereditary transthyretin amyloidosis	- Norfolk QoL-DN<br>**- EQ-5D-5L**	**- EQ-5D data collected in the clinical trials**
HST11[#]	Voretigene neparvovec for treating inherited retinal dystrophies caused by RPE65 gene mutations	- Visual function questionnaire	- Literature/ other studies
HST12	Cerliponase alfa for treating neuronal ceroid lipofuscinosis type 2	- PedsQL Parent Report for Toddlers<br>- PedsQL family impact module (PedsQL-FIM)<br>- CLN2-specific quality-of-life instrument<br>- **EQ-5D-5L [collected, but not used for the QALY calculation]**	- Vignette study
HST13	Volanesorsen for treating familial chylomicronaemia syndrome	- SF-36<br>- **EQ-5D [collected, but not used for the QALY calculation]**	- Vignette study
HST14	Metreleptin for treating lipodystrophy	- No QoL data collected	- Vignette study

HST15[#]	Onasemnogene abeparvovec for treating spinal muscular atrophy	- No QoL data collected	- Literature/ other studies
HST16	Givosiran for treating acute hepatic porphyria	-**EQ-5D [collected, but not used for the QALY calculation]**	- Literature/ other studies, proxy condition
HST17	Odevixibat for treating progressive familial intrahepatic cholestasis	- PedsQL	- Vignette study
HST18[#]	Atidarsagene autotemcel for treating metachromatic leukodystrophy	- No EQ-5D data collected	- Vignette study
HST19	Elosulfase alfa for treating mucopolysaccharidosis type 4A (review of HST2)	**- EQ-5D-5L collected as part of the managed access agreement**	- **EQ-5D-5L collected as part of the managed access agreement**<br>- Additional sources
HST20	Selumetinib for treating symptomatic and inoperable plexiform neurofibromas associated with type 1 neurofibromatosis in children aged 3 and over	- Paediatric Quality of Life Inventory (PedsQL)	- Vignette study
HST21	Setmelanotide for treating obesity caused by LEPR or POMC deficiency	- Impact of Weight on Quality of Life (IWQOL)-Lite instrument (in adults)<br>- Paediatric Quality of Life Inventory (PedsQL)	- Vignette study

HST: Highly specialised technology; SF-36: Short Form-36 questionnaire.

* Non-inferiority trials testing the standard of care against new oral formulations assessed as having the same efficacy.

[#] Gene therapies

Table S3: Time horizons of previously assessed highly specialised technologies

Time horizons for highly specialised technologies with concluded appraisals (until December 2022), as reported in the Final Evaluation Determination documents available from the NICE homepage.

HST	Time horizon [years]	Source
HST1	Lifetime time horizon, 125 y. [sic!]	- NICE (2015): Final Evaluation Determination document, p.10 ¶ 4.18 https://www.nice.org.uk/guidance/hst1/documents/atypical-haemolytic-uraemic-syndrome-ahus-eculizumab-final-evaluation-determination3<br>- Tappenden P, Bessey A, Pandor A, Kaltenthaler E, Cantrell A, Harvey B, Hernandez M, Richards A, Inward C, Saleem M. Eculizumab for treating atypical haemolytic uraemic syndrome. Final report to the National Institute for Health and Care Excellence, 2013., p.77
HST2	Lifetime time horizon, 100 y.	NICE (2015): Final Evaluation Determination document, p.10 ¶ 4.11 (https://www.nice.org.uk/guidance/hst2)
HST3	Lifetime time horizon, 35 y.	- NICE (2016): Final Evaluation Determination document, p.43 https://www.nice.org.uk/guidance/hst3/documents/final-evaluation-determination-document<br>- Ataluren for treating Duchenne muscular dystrophy with a nonsense mutation in the dystrophin gene. A Highly Specialised Technology. Warwick Evidence, August 2015., p.123
HST4*	Lifetime time horizon, 48 y.	NICE (2016): Final Evaluation Determination document, p.7 ¶ 4.11 https://www.nice.org.uk/guidance/hst4/documents/final-evaluation-determination-document
HST5*	70 y.	NICE (2017): Final Evaluation Determination document, p.14 ¶ 4.31 https://www.nice.org.uk/guidance/hst5/documents/final-evaluation-determination-document<br>“The starting age of people in the treatment-naive population was assumed to be 32 years based on the mean age in the ENGAGE trial. The starting age of people in the population whose disease was stable with ERT who

		switched to eliglustat was assumed to be 38 years." P.14 ¶4.29
HST6	Lifetime time horizon	NICE (2017): Final Evaluation Determination document, p.12 ¶ 4.19 https://www.nice.org.uk/guidance/hst6/documents/final-evaluation-determination-document-2
HST7[#]	Lifetime time horizon, 100 y.	NICE (2017): Final Evaluation Determination document, p.14 ¶ 4.19 https://www.nice.org.uk/guidance/hst7/documents/final-evaluation-determination-document (NICE 2017): Committee papers, p.565 https://www.nice.org.uk/guidance/hst7/documents/committee-papers
HST8	Lifetime time horizon	NICE (2018): Final Evaluation Determination document, p.19-20 ¶ 4.23 https://www.nice.org.uk/guidance/hst8/documents/final-evaluation-determination-document
HST9	Lifetime time horizon, 41 y.	NICE (2018): Final Evaluation Determination document, p.19-11 ¶ 4.12 https://www.nice.org.uk/guidance/hst9/documents/final-evaluation-determination-document However, it considered that this criterion unfairly penalises people with hATTR amyloidosis because they are older and so would have a life expectancy of less than 30 years even without this condition, p.18 ¶ 4.25
HST10	Lifetime time horizon, 40 y.	(NICE 2018): Committee papers, p.214 https://www.nice.org.uk/guidance/hst10/documents/committee-papers
HST11[#]	Lifetime time horizon, 100 y.	NICE (2019): Committee Papers, p. 251 https://www.nice.org.uk/guidance/hst11/documents/committee-papers-2
HST12	Lifetime time horizon, 95 y.	NICE (2018): Committee Papers, p. 609 https://www.nice.org.uk/guidance/hst12/documents/committee-papers
HST13	59 y.	NICE (2020): Final Evaluation Determination document, p.16 ¶ 4.18 "The company's economic analysis adopted an NHS perspective and implemented a 59-year time horizon (assumed to represent the maximum remaining lifetime of a patient)." https://www.nice.org.uk/guidance/hst13/documents/final-evaluation-determination-document

HST14	Lifetime horizon; 100 y.	NICE (2021): Committee Papers., p. 704 https://www.nice.org.uk/guidance/hst14/documents/committee-papers-2
HST15#	Lifetime horizon	NICE (2021): Final Evaluation Determination document, p.30 ¶ 4.32 https://www.nice.org.uk/guidance/hst15/documents/final-evaluation-determination-document
HST16	60 y.	NICE (2021): Final Evaluation Determination document, p.11 ¶ 4.14 https://www.nice.org.uk/guidance/hst16/documents/final-evaluation-determination-document “ […] treatment starting age of 37 within modelling”, p.20 ¶ 4.31
HST17	Lifetime time horizon, 100 y.	NICE (2018): Committee papers, p. 180 https://www.nice.org.uk/guidance/hst17/documents/committee-papers
HST18#	Lifetime horizon, 100 y.	NICE (2022): Committee papers, p. 688 https://www.nice.org.uk/guidance/hst18/documents/committee-papers
HST19, review HST2	Lifetime horizon, 100 y.	NICE (2021): Committee papers, p. 870 https://www.nice.org.uk/guidance/hst19/documents/committee-papers
HST20	Lifetime horizon, 100 y.	NICE (2021): Committee papers, p. 180 https://www.nice.org.uk/guidance/hst20/documents/committee-papers
HST21	Lifetime horizon, 100 y.	NICE (2022): Committee papers, p. 456 https://www.nice.org.uk/guidance/hst21/documents/committee-papers

HST: Highly specialised technology

* Non-inferiority trials testing the standard of care against new oral formulations assessed as having the same efficacy.

Gene therapies

Table S4: Discount rates and QALY gains of previously assessed highly specialised technologies

Discount rates and QALY gains for highly specialised technologies with concluded appraisals (until December 2022), as reported in the Final Evaluation Determination documents available from the NICE homepage. Since 2017, highly specialised technologies that produce an undiscounted QALY gain between 10 to 30 QALYs over the lifetime of a patient are considered highly effective and are multiplied (weighted) by a factor between one to maximum three. Some of the QALY gains are only reported by NICE as ranges, for example ≥ 10, because they are considered commercial in confidence by the manufacturers.

A: Discount rates and QALY gains of previously assessed highly specialised technologies, technologies tested in non-inferiority trials

Non-inferiority trials testing the standard of care (enzyme replacement therapy) against new oral formulations assessed as having the same efficacy.

HST	Discount rate	QALY gains undiscounted	QALY gains discounted	Weighting
HST4	3.5%		0.34	n.a. (evaluated before 2017)
HST5	3.5%		ERT stable: 1.05<br>ERT naïve: 1.06	n.a. (evaluated before 2017)

QALY: Quality Adjusted Life Year; HST: Highly specialised technology; ERT: Enzyme replacement therapy

B: Discount rates and QALY gains of previously assessed highly specialised technologies, gene therapies

HST	Discount rate	QALY gains undiscounted	QALY gains discounted	Weighting
HST7	1.5% and 3.5%	14		Applied, factor 1.4
HST11	3.5%	12		Applied, factor 1.2
HST15	1.5%	18.62		Partially applied, factor not given
HST18	3.5%	≥ 10 to ≥ 30		Applied, factor between 1 to 3

QALY: Quality Adjusted Life Year; HST: Highly specialised technology

C: Discount rates and QALY gains of previously assessed highly specialised technologies, other technologies

HST	Discount rate	QALY gains undiscounted	QALY gains discounted	Weighting
HST1	1.5%		10.14	n.a. (evaluated before 2017)
HST2	1.5%		5.04	n.a. (evaluated before 2017)
HST3	3.5%		> 3.05	n.a. (evaluated before 2017)
HST6	3.5%		Overall population: 14.13<br>Subgroups<br>- Perinatal and infantile onset: 15.6<br>- Juvenile onset:<br>0-4 years: 14.7<br>5-11 years: 14.2<br>12-17 years: 14.1<br>≥ 18 years: 9.8	n.a. (evaluated before 2017)
HST8	3.5%	≥ 10 to 15.99		Applied, factor not given
HST9	3.5%	< 10		Not applied
HST10	3.5%	< 10		Not applied
HST12	3.5%	≥ 30		Applied, factor 3
HST13	3.5%	< 10		Not applied
HST14	3.5%	< 10		Not applied
HST16	3.5%	18.6		Applied, factor 1.8
HST17	3.5%	< 10		Not applied
HST19	3.5%	≥ 10		Applied, factor not given
HST20	3.5%	< 10		Not applied
HST21	3.5%	> 10		Applied, factor not given

QALY: Quality Adjusted Life Year; HST: Highly specialised technology

Table S5: Information for the back-calculation for the QALY gains, afamelanotide for treating erythropoietic protoporphyria [ID927]

QALY gains in the evaluation of afamelanotide for treating erythropoietic protoporphyria at the National Institute for Health and Care Excellence (NICE) in England. Link: https://www.nice.org.uk/guidance/indevelopment/gid-hst10009

ID927	Initial assessment (2018)	Current assessment (2022)
(1) QoL data used for the QALY calculation	Final Evaluation Determination document (May 2018), p.16 ¶ 4.16 https://www.nice.org.uk/guidance/gid-hst10009/documents/final-evaluation-determination-document	Evaluation Consultation Document (September 2022), p.33 ¶ 4.46 https://www.nice.org.uk/guidance/gid-hst10009/documents/evaluation-consultation-document-3
(2) Utilities	Calculated from (3) and (5)	Calculated from (3) and (5)
(3) Assumed time horizon	Committee papers (issued 20 Dec 2017), p.444 https://www.nice.org.uk/guidance/gid-hst10009/documents/committee-papers	Evaluation Consultation Document (September 2022), p.33 ¶ 4.46 https://www.nice.org.uk/guidance/gid-hst10009/documents/evaluation-consultation-document-3
(4) Discount rate	Committee papers (issued 20 Dec 2017), p.74 https://www.nice.org.uk/guidance/gid-hst10009/documents/committee-papers	Standard rate (3.5%) applied
(5) QALY gain undiscounted	Final Evaluation Determination document (May 2018), p.18 ¶ 4.19 https://www.nice.org.uk/guidance/gid-hst10009/documents/final-evaluation-determination-document	Calculated from (3), (7), (8) and (9)

(6) QALY gain discounted	Calculated from (4) and (5)	Calculated from (2), (3) and (4)
(7) Cost per dose	Final Evaluation Determination document (May 2018), p.3 ¶ 3.3 https://www.nice.org.uk/guidance/gid-hst10009/documents/final-evaluation-determination-document	Evaluation Consultation Document (September 2022), p.5 ¶ 3.3 https://www.nice.org.uk/guidance/gid-hst10009/documents/evaluation-consultation-document-3
(8) Number of doses per year	Final Evaluation Determination document (May 2018), p.3 ¶ 3.1 https://www.nice.org.uk/guidance/gid-hst10009/documents/final-evaluation-determination-document	Evaluation Consultation Document (September 2022), p.17 ¶ 4.18 https://www.nice.org.uk/guidance/gid-hst10009/documents/evaluation-consultation-document-3
(9) ICER(s)	Final Evaluation Determination document (May 2018), p.19 ¶ 4.20 https://www.nice.org.uk/guidance/gid-hst10009/documents/final-evaluation-determination-document	Evaluation Consultation Document (September 2022), p.39 ¶ 4.58 https://www.nice.org.uk/guidance/gid-hst10009/documents/evaluation-consultation-document-3

QALY: Quality Adjusted Life Year; HST: Highly specialised technology, ICER: Incremental cost-effectiveness ratio.
